# Electrochemical Properties of Lipid Membranes Self-Assembled from Bicelles

**DOI:** 10.3390/membranes11010011

**Published:** 2020-12-23

**Authors:** Damian Dziubak, Kamil Strzelak, Slawomir Sek

**Affiliations:** 1 Faculty of Chemistry, Biological and Chemical Research Centre, University of Warsaw, Żwirki i Wigury 101, 02-089 Warsaw, Poland; slasek@chem.uw.edu.pl; 2 Faculty of Chemistry, University of Warsaw, Pasteura 1, 02-093 Warsaw, Poland; kamil.strzelak@chem.uw.edu.pl

**Keywords:** electrochemistry, gold electrode, supported lipid membranes, bicelles, self-assembly

## Abstract

Supported lipid membranes are widely used platforms which serve as simplified models of cell membranes. Among numerous methods used for preparation of planar lipid films, self-assembly of bicelles appears to be promising strategy. Therefore, in this paper we have examined the mechanism of formation and the electrochemical properties of lipid films deposited onto thioglucose-modified gold electrodes from bicellar mixtures. It was found that adsorption of the bicelles occurs by replacement of interfacial water and it leads to formation of a double bilayer structure on the electrode surface. The resulting lipid assembly contains numerous defects and pinholes which affect the permeability of the membrane for ions and water. Significant improvement in morphology and electrochemical characteristics is achieved upon freeze–thaw treatment of the deposited membrane. The lipid assembly is rearranged to single bilayer configuration with locally occurring patches of the second bilayer, and the number of pinholes is substantially decreased. Electrochemical characterization of the lipid membrane after freeze–thaw treatment demonstrated that its permeability for ions and water is significantly reduced, which was manifested by the relatively high value of the membrane resistance.

## 1. Introduction

Lipid bilayers supported on solid substrates are considered as an important model to mimic the natural cell membranes in fundamental studies [1,2,3,4]. These systems were also proven to be suitable for the construction of biosensors and bioanalytical platforms for the examination of membrane proteins [5,6,7]. The immobilization of the membrane at the supporting substrate offers a unique opportunity to probe the properties of such an assembly with numerous surface-sensitive techniques. These include scanning probe microscopy, infrared reflective absorption spectroscopy, quartz crystal microbalance, and for conductive supports, electrochemical methods can be used as well [8,9,10,11]. Most popular approaches for supported lipid membrane formation involve vesicles spreading or Langmuir–Blodgett and Langmuir–Schafer techniques [12,13,14,15]. It was demonstrated in numerous research papers that both can produce well-defined planar bilayers with good electrical insulating properties manifested by low differential capacitance and high membrane resistance [7,16]. This issue is of crucial importance for the biosensors, which are sensitive to the structural or functional changes of lipid assemblies triggered by membrane proteins, or the constructs, which may act as affinity sensors detecting interactions of biological material with lipid membrane [17,18]. In particular, the modulation of the ion permeability of lipid membranes may be utilized in biosensors or the studies of pore-forming toxins since the dielectric damage can be transduced into the physical signal utilizing electrochemical methods [16].

An alternative approach was proposed involving the formation of supported lipid membranes from bicellar mixtures. Bicelles are composed of long-chain and short-chain phospholipids and tend to form disk-like aggregates [19,20]. Such lipidic assemblies are broadly utilized in structural biology studies due to their ability to host membrane proteins while retaining protein structure and function [21]. The planar bilayer formation on silicon chips from a bicellar mixture of 1,2-dipalmitoyl-*sn*-glycero-3-phosphocholine and 1,2-diheptanoyl-*sn*-glycero-3-phosphocholine lipids was first reported by Zeineldin and coworkers [22]. The optimal routes to fabricate supported lipid bilayers from bicelles were later demonstrated by Cho’s group, who also revealed mechanistic details of the lipid membrane formation on hydrophilic substrates such as silicon dioxide, titanium oxide, and aluminum oxide [23,24]. These authors have shown that the adsorption behavior of bicelles can vary depending on the nature of the supporting surface, and the electrostatic attraction between the surface and adsorbing bicelles is necessary for the successful formation of the supported lipid bilayer (SLB). A similar conclusion can be drawn based on the results reported by Yamada and coworkers, who utilized atomic force microscopy and force spectroscopy to probe the properties of lipid films assembled from bicelles [25]. It was found that lamellae of phospholipid bilayers were aligned parallel to a surface in case of the negatively charged bare silicon substrate, while unoriented phospholipid bilayers were formed on Si substrate modified with terminal amine groups, where the excess of positive surface charge is expected.

In this work, we have described the mechanism of bicelles adsorption onto thioglucose-modified gold electrodes. The architecture of the resulting membrane can be considered as a floating lipid membrane, which is separated from the substrate by a monolayer of hydrophilic molecules of thioglucose [26,27]. In such a configuration, the polar heads of lipids located close to the electrode surface remain hydrated and the direct interaction with metal is eliminated. Immobilization of the lipid assembly on conductive support enabled electrochemical characterization of the resulting membrane and assessment of its permeability for ions. Additionally, the effect of freeze–thaw treatment on membrane electrical insulating properties and morphology was investigated.

## 2. Materials and Methods

Chemicals. 1,2-dimyristoyl-*sn*-glycero-3-phosphocholine (DMPC) and 3-([3-cholamidopropyl]dimethylammonio)-2-hydroxy-1-propanesulfonate (CHAPSO) were purchased from Avanti Polar Lipids Inc., Alabaster, AL, USA. Sodium fluoride, 1-thio-β-D-glucose, hydrofluoric acid, and sodium tetrachloroaurate were purchased from Sigma-Aldrich Sp. z. o. o., Poznan, Poland. All other reagents and solvents were obtained from Avantor Performance Materials Poland S.A., Gliwice, Poland. All chemicals were used as received. The water was purified through the Milli-Q system (resistivity 18.2 MΩ × cm). In all experiments, we have used an aqueous solution of 0.1 M NaF.

Bicelles preparation. Bicelles were prepared according to the protocol described by Ujwal and coworkers [28]. 260 mg of DMPC and 90 mg of CHAPSO were transferred to the vial and mixed with 1 mL of ultrapure Milli-Q water with a resistivity of 18.2 MΩ × cm. To disperse lipids and obtain bicelles, the suspension was warmed to 40 °C and then cooled to −20 °C. The steps with warming and cooling were repeated more than 25 times until the mixture at room temperature was homogeneous and in gel consistency. However, 10 cycles should be enough to observe the formation of the gel at room temperature and cloudiness upon cooling. For the experiments, bicelles were dissolved in 0.1 M NaF solution with a volume ratio of 1:1000.

Electrochemistry. Electrochemical experiments were performed using CHI 650B potentiostat (CH Instruments Inc., Austin, TX, USA) in a three-electrode cell with an Ag|AgCl|sat.KCl reference electrode, a Pt foil counter electrode, and an Au(111) working electrode. The measurements were carried out in a hanging meniscus configuration. Before each experiment, the Au(111) working electrode was cleaned in piranha solution (H_2_SO_4_: H_2_O_2_ 3:1 *v*/*v*. CAUTION: piranha reacts violently with organic compounds) for at least 12 h. Then it was thoroughly rinsed with ultrapure water and flame annealed. The monolayers of 1-thio-β-D-glucose (further referenced as thioglucose) were obtained by immersing the Au(111) electrode in an aqueous solution containing 0.1 mg/mL of the thioglucose for ~2 h. Next, the electrodes were rinsed with ultrapure water and immersed in bicellar suspension for at least 3 h to obtain lipid assembly. For the freeze–thaw treatment, the electrode was taken out from the suspension and covered with a thin film of the supporting electrolyte. Freeze–thaw treatment was carried out by cooling the electrode with deposited lipid film down to −18 °C for 2 h and then warming it up to room temperature. A single freeze–thaw cycle was performed for each experiment. AC voltammetry measurements were performed with a scan rate of 5 mV/s. The RMS amplitude of AC perturbation was 10 mV, and a frequency set at 20 Hz. The differential capacitance was derived based on AC voltammograms from the in-phase and out-of-phase components of the AC signal under the assumption that the electrode-electrolyte interface can be treated as a simple RC circuit. Electrochemical impedance spectroscopy (EIS) measurements were carried out within the frequency range of 10^−1^ to 10^4^ Hz and the amplitude AC perturbation was 10 mV. All measurements were carried at 22 ± 1 °C. The potentials reported in this work are referenced to the Ag|AgCl|sat.KCl electrode.

Topography Imaging. Atomic force microscopy (AFM) experiments were performed with Dimension Icon (Bruker Corporation, Billerica, MA, USA). The imaging of the samples was performed using ScanAsyst Fluid probes (Bruker, nominal spring constant 0.7 N/m, tip radius ~20 nm) in PeakForce Tapping mode. The cantilever was periodically modulated with default amplitude at the frequency of 2 kHz. The exact value of the spring constant and the deflection sensitivity for a given probe was carefully calibrated by thermal tuning before each experiment. All images were recorded in an aqueous solution of 0.1 M NaF at the temperature of 22 ± 1 °C. In situ imaging was performed on an Au(111) single crystal (MaTecK, GmbH, Julich, Germany) pre-modified with thioglucose monolayer. The quality of the bare Au(111) surface was verified by AFM imaging before each experiment (see Figure S1 in Supplementary Materials). The images were processed using Nanoscope Analysis software version 1.40 (Bruker Corporation, Billerica, MA, USA) and involved flattening with a first-order polynomial function. The protocol of the thioglucose immobilization on gold was the same as for electrochemical measurements. The images were collected immediately after injection of bicellar suspension into the liquid cell of the atomic force microscope. Freeze–thaw treatment was performed by cooling the sample with deposited lipid film down to −18 °C and then warming it up to room temperature.

Surface Enhanced Infrared Absorption Spectroscopy. The spectra were recorded with Nicolet iS50 FTIR spectrometer (Thermo Fisher Scientific, Waltham, MA, USA) with MCT-A detector and custom-made single-reflection accessory. The incident angle was 60° and the spectral resolution was 4 cm^−1^. The all-glass custom-made spectroelectrochemical cell was used in all experiments with platinum foil serving as a counter electrode and Ag|AgCl|sat.KCl as a reference electrode. The working electrode was a thin gold film deposited on a reflectance plane of a Si hemispherical prism. Deposition of gold was carried out by dropping an aqueous plating solution onto the hydrogen-terminated Si surface. The plating solution was obtained by mixing 100 μL of 0.03 M NaAuCl_4_·2H_2_O, 2 mL of 0.15 M Na_2_SO_3_ + 0.05 M Na_2_S_2_O_3_·5H_2_O + 0.05 M NH_4_Cl, and 1 mL of 2% HF. After approximately 90 s of plating, the prism was rinsed with ultrapure water to finish the deposition. The deposited film was further modified by dropping an aqueous solution of thioglucose (0.1 mg/mL) onto the gold surface and after 2 h the prism was gently rinsed with ultrapure water. The spectra are displayed in absorbance units defined as A = log(*I*_0_/*I*), where *I*_0_ corresponds to the intensities of IR radiation observed for the reference spectra, while *I* corresponds to the intensity observed for the sample. The reference spectrum was collected for gold film modified with thioglucose. Data processing was performed using Omnic 9 software (Thermo Fisher Scientific, Waltham, MA, USA).

## 3. Results and Discussion

**Figure 1:**
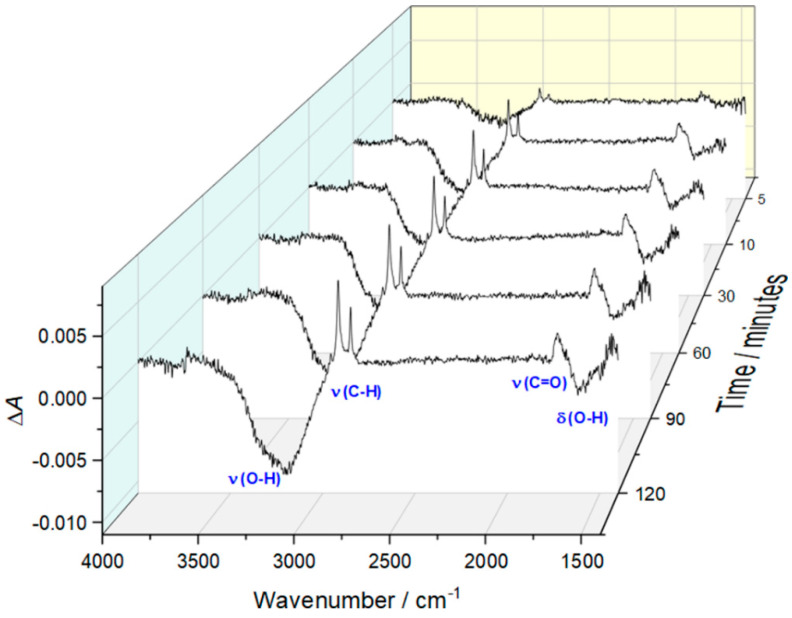
Time-evolution of surface-enhanced infrared absorption (SEIRA) spectra collected for thioglucose-modified gold during the deposition of bicelles. The reference spectrum was collected for gold film modified with monolayer of thioglucose.

Self-assembly of bicelles on thioglucose-modified gold was monitored using surface-enhanced infrared absorption spectroscopy (SEIRAS). In this technique, the infrared absorption intensity can be significantly enhanced by 10–1000 times on coinage metal nanoparticles [29]. The electromagnetic field of the incident light induces an oscillating dipole in the metal nanoparticle by excitation of the localized plasmon. The induced dipole produces an electric field in the vicinity of the nanoparticle and excites the absorbed molecules. The electric field is normal to the metal surface, which means that molecular vibrations with the component of the transition dipole moment normal to the local surface can be excited and enhanced. Moreover, the local electric field decays within a short distance away from the surface. Since the dominant contribution comes from the species close to the metal surface, the adsorption and desorption processes can be monitored using this technique. Figure 1 illustrates the time evolution of the spectra recorded during DMPC-CHAPSO bicelles deposition. The spectra were collected at open circuit potential, and each represents the difference in absorbance between the spectrum at the specified time and the spectrum recorded as reference. The latter was collected for thioglucose-modified gold film deposited on a silicon prism before the addition of bicellar suspension. The most pronounced changes occur in the spectral region corresponding to the O–H stretching between 3000 and 3600 cm^−1^. The absolute intensity of the negative band increases regularly within the time, demonstrating that the amount of water in the interfacial region decreases gradually. The minimum of the *v*(O–H) band is located at ~3225 cm^−1^ and it contains a visible shoulder at ~3350 cm^−1^. The ν(O–H) bands within this spectral region correspond to the water molecules in a network of hydrogen bonds [30,31,32]. Hence, the emergence of the negative ν(O–H) band indicates that the adsorption of bicelles involves the replacement of hydrogen-bonded water from the interfacial region. This is confirmed by the presence of the negative δ(O–H) band at ~1630 cm^−1^, which can also be ascribed to hydrogen-bonded water.

In parallel with the loss of interfacial water, an increase in the intensity of the positive bands corresponding to ν(C–H) and ester ν(C=O) vibrations is observed. Both are related to the adsorption of lipid molecules on the electrode surface. The bands associated with ν_as_(C–H) and ν_s_(C–H) vibrations are located at 2917 cm^−1^ and 2849 cm^−1^, respectively, which indicates that the lipids forming the assembly are in the gel state with the acyl chains adopting all-trans conformation [33]. This observation is understandable since the experiments were performed at 22 °C and the main gel (*L_β_*) to liquid–crystalline (*L_α_*) phase transition of DMPC occurs at ~23–24 °C. Importantly, the position of the ester ν(C=O) band at ~1725 cm^−1^ is indicative of hydrogen-bonded populations of ester groups that are fully hydrated [34]. These experimental observations demonstrate that bicelles are successfully deposited on thioglucose-modified gold, and the effect of the substrate on lipid conformation and hydration of the polar heads is rather small.

Further evaluation of bicelles adsorption was performed using in situ atomic force microscopy (AFM). This method has a unique capability to image the topography of the surface films in nanoscale and to provide information about their thickness. Importantly, the imaging can be performed under in situ conditions, therefore numerous surface-related processes can be monitored in real-time [11,35,36].

**Figure 2:**
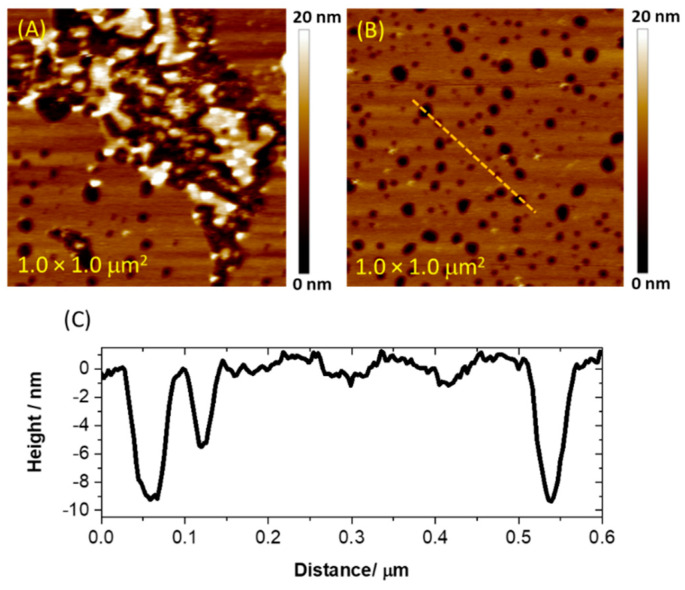
Atomic force microscopy (AFM) images collected for thioglucose-modified Au(111) after (**A**) 10 min and (**B**) 90 min of deposition of bicelles. Panel (**C**) presents the cross-sectional profile for the completed lipid assembly. The profile was taken along the orange dotted line displayed in panel (**B**). The images were collected in Peak Force Tapping mode.

Figure 2 presents the images of a thioglucose-modified Au(111) electrode exposed to bicellar suspension. Within approximately 10 min upon injection of the bicelles, the electrode surface is covered with patches of lipidic material with the height varying within the range of ~10–15 nm. Since the expected thickness of the single bilayer formed by DMPC is ~4–5 nm, this indicates that bicelles tend to adsorb as stacks and form double or triple bilayers. Time-lapse imaging revealed that randomly shaped lipidic deposits further grow and merge into the stable planar film with numerous pinholes. The total thickness of the film measured from cross-sectional profiles taken along large defect sites was found to be 9.5 ± 0.4 nm, indicating that the lipid assembly is a double bilayer, and the defects span the entire thickness of the film. However, the depth of some fraction of the smaller pinholes was 4.8 ± 0.4 nm (see Figure 2C). In such a case, only the top bilayer is perforated, while the bottom film maintains continuity. Thus, the AFM imaging confirmed successful adsorption of bicelles on a thioglucose-modified gold surface. However, the resulting film does not adopt a single bilayer configuration, and it contains numerous defects spanning either the entire film or only the top bilayer.

**Figure 3:**
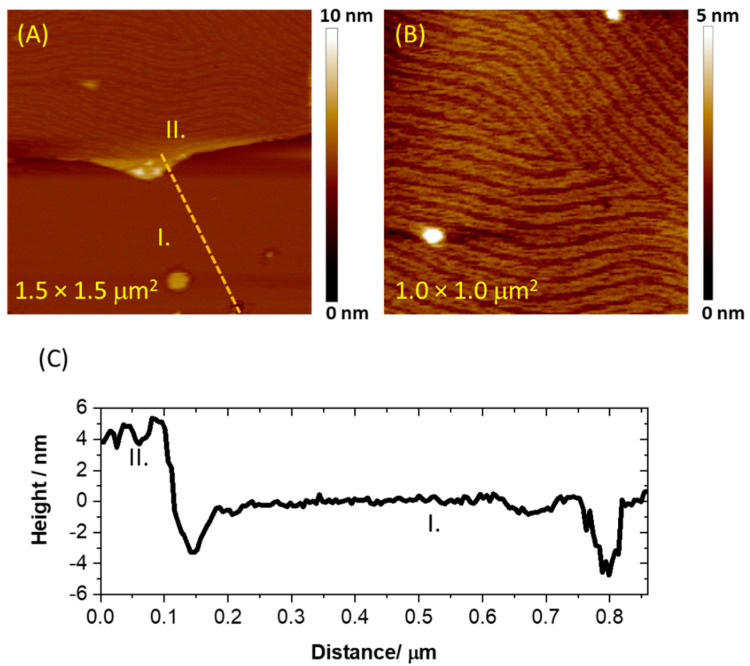
(**A**) An AFM image collected for lipid assembly deposited on thioglucose-modified Au(111) after one cycle of freeze–thaw treatment. (**B**) An AFM image of the double bilayer region with ripple phase structure. (**C**) The cross-sectional profile for lipid assembly after one freeze–thaw cycle. The profile was taken along the orange dotted line displayed in panel (**A**). The images were collected in Peak Force Tapping mode.

To improve the properties of the lipid assembly, we have subjected the samples to freeze–thaw cycle. Such an approach assumes that osmotic stress during freezing and thawing can lead to the destabilization and mechanical rupture of lipid membranes, which in our case might induce significant membrane reorganization. Moreover, the previous AFM studies have demonstrated that interfacial water in phosphatidylcholine lipid bilayer is ordered at room temperature and the ions act as bridges between the lipid polar heads [37,38]. However, when the temperature is lowered below 4 °C, the order of the hydration shell of the bilayer might decrease due to the negative thermal expansion coefficient of water. This is manifested by the substantial decrease of Young’s modulus of the bilayer, which could be related to increased efflux of bridging ions and the creation of a thicker and more disordered interface. As a result, the distance between polar heads is increased, which could make the assembly more prone to reorganization. The effect of the freeze–thaw cycle on the topography of the lipid film is shown in Figure 3.

In this case, the number of the pinholes is significantly reduced, and the large area of the electrode is covered with continuous film represented by region I in Figure 3A. Its thickness was found to be 4.7 ± 0.3 nm, proving that a single bilayer is formed upon the freeze–thaw cycle. Nevertheless, the presence of the double bilayer is still observed locally, which is represented by region II in Figure 3A. Interestingly, the top bilayer in region II exhibits corrugation with well-defined periodicity typical for the ripple phase (P_β’_) [39,40]. Figure 3B shows a more detailed image of this structure, and it can be observed that the corrugations form long ripples composed of twinned stripes. The spacing between the neighboring ripples is 54 ± 10 nm, while the distance between twinned lines is 15 ± 3 nm (see Figure S2 in Supplementary Materials). The average amplitude of the ripples was found to be 0.92 ± 0.18 nm. The ripple phase is known to exist in a temperature range between the pretransition and the main phase transition, and it is usually observed for multibilayer phospholipid assemblies supported on a solid surface [40]. Importantly, the repetitive AFM imaging of the samples stored at 22 °C under the electrolyte solution revealed that the morphology of the lipid film does not change significantly within one week. The only visible change was related to the gradual disappearance of the ripple phase.

**Figure 4:**
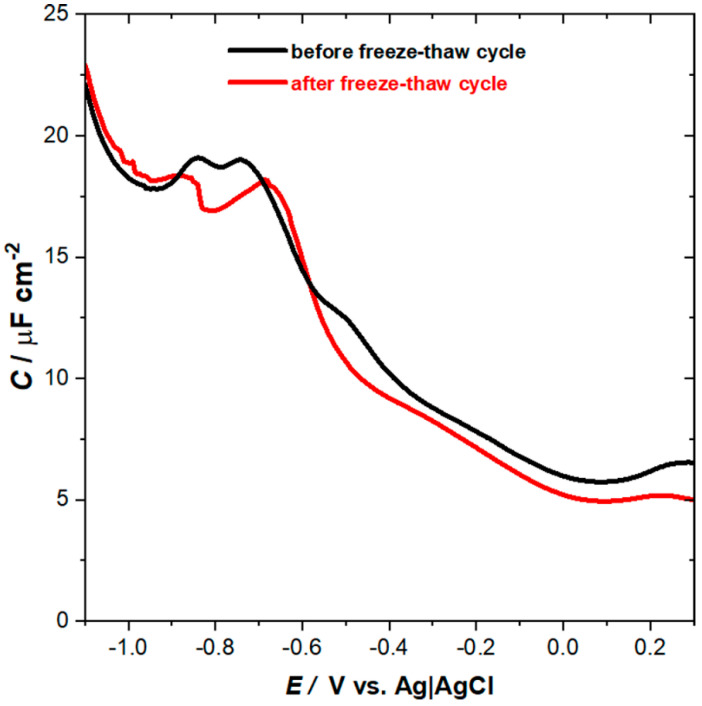
Potential dependence of differential capacitance of the thioglucose-modified Au(111) electrode with lipid film before (black) and after (red) one freeze–thaw cycle.

Hence, the AFM data demonstrate clearly that the perforated double bilayer formed by bicelles deposition on thioglucose-modified gold can be effectively changed by the freeze–thaw procedure. The latter leads to the formation of a single bilayer coexisting with a double bilayer, and the number of pinholes is significantly reduced. Such a transformation should affect the electrochemical behavior of the lipid film. Therefore, we have used AC voltammetry and electrochemical impedance spectroscopy to further investigate the properties of the lipid membranes. Figure 4 illustrates the changes in a differential capacitance as a function of the potential recorded for thioglucose-modified gold electrodes with deposited lipid film.

**Figure 5:**
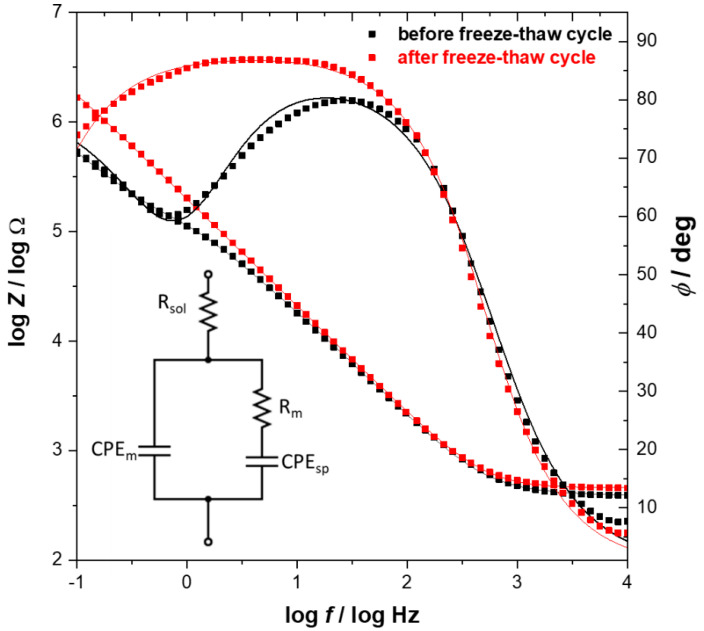
Bode plot obtained for the thioglucose-modified Au(111) electrode with lipid film before (black) and after (red) one freeze-thaw cycle. Solid lines represent the fit based on the equivalent circuit shown as an inset, where *R*_sol_ is the resistance of the electrolyte, *R*_m_ and *CPE*_m_ are the resistance and constant phase element of the lipid membrane, and *CPE*_sp_ represents the constant phase element of the spacer region between the gold surface and lipid film.

Potential-dependent changes in differential capacitance are quite similar before and after freeze–thaw treatment. The lipid film is most stable between +0.2 V and −0.1 V, and a well-pronounced minimum of the capacitance is observed at +0.1 V. Negative polarization of the electrode causes an increase of the capacitance, which is often explained by electroporation and/or lifting the membrane leading to accumulation of water and ions at the interfacial region [31,41]. At the potential of −0.6 V, the pseudocapacitive peaks are observed reflecting the desorption of the lipid film from the electrode, which is then followed by reductive desorption of thioglucose monolayer at −0.8 V. Although, the observed changes are qualitatively similar, it is evident that the properties of the lipid film that after freeze–thaw cycle are improved, as it is manifested by an overall decrease of the measured capacitance. Referring to AFM data, this effect can be ascribed to fewer pinholes and defect sites after freeze–thaw treatment. Further verification of the electrical properties of the lipid membrane assembled from bicelles was carried out using electrochemical impedance spectroscopy (EIS). The resulting Bode plots are shown in Figure 5. The spectra were collected at the potential of +0.1 V, where a minimum in differential capacitance is observed and the lipid membrane is most stable.

**Figure 6:**
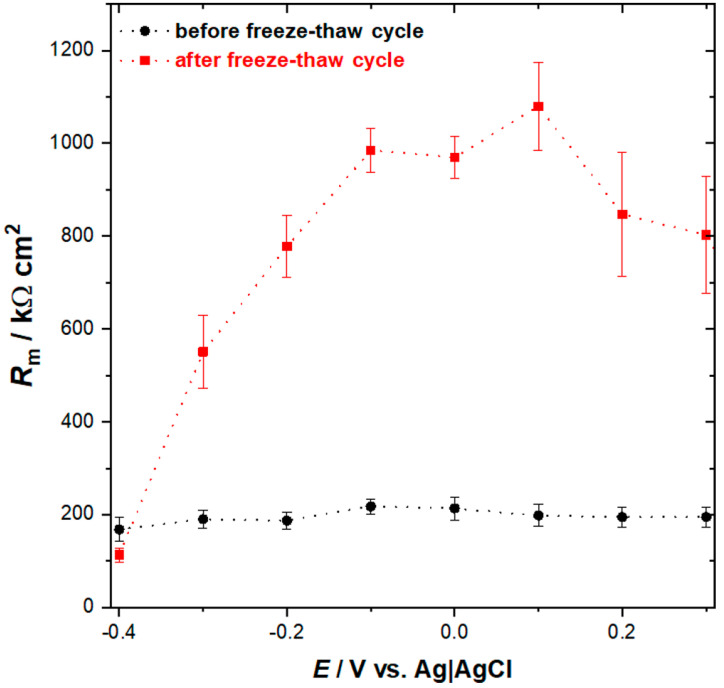
Changes of the membrane resistance (*R*_m_) as a function of the potential applied to the thioglucose-modified Au(111) electrode with lipid assembly before (black) and after (red) one freeze–thaw cycle.

The EIS spectra obtained for lipid film before freeze–thaw treatment display features characteristics for defected membranes. Namely, the presence of defects is manifested by a steplike feature on the total impedance curve and pronounced phase minimum observed at frequency ~1 Hz. However, the phase angle minimum of ~60° is quite shallow, which may indicate the heterogeneous distribution of defects within the membrane [42]. Such interpretation is supported by AFM results where numerous randomly distributed pinholes of different sizes were observed. The EIS spectra for lipid film after the freeze–thaw cycle demonstrates that the total impedance is higher, and the phase angle plot displays a plateau within the frequency range of 1–100 Hz, corresponding to a value ~85°. This indicates that the impedance is predominantly determined by capacitance. At lower frequencies, the decrease of the phase angle is observed, which suggests that some defects are still present, but according to the model developed by Valincius and coworkers, it can be concluded that their density is much lower compared with the membrane before freeze-thaw treatment [43]. More quantitative analysis of the electrical properties of lipid films assembled from bicelles can be performed by comparison of the membrane resistance (*R*_m_), which reflects the membrane permeability for water and ions. For this purpose, the EIS data were fitted with the equivalent circuit shown as an inset in Figure 5. This model assumes that the lipid membrane is separated from the electrode surface by a hydrophilic spacer and enables the estimation of membrane resistance. The plot illustrating the changes in values of *R*_m_ as a function of the potential is shown in Figure 6.

**Figure 7:**
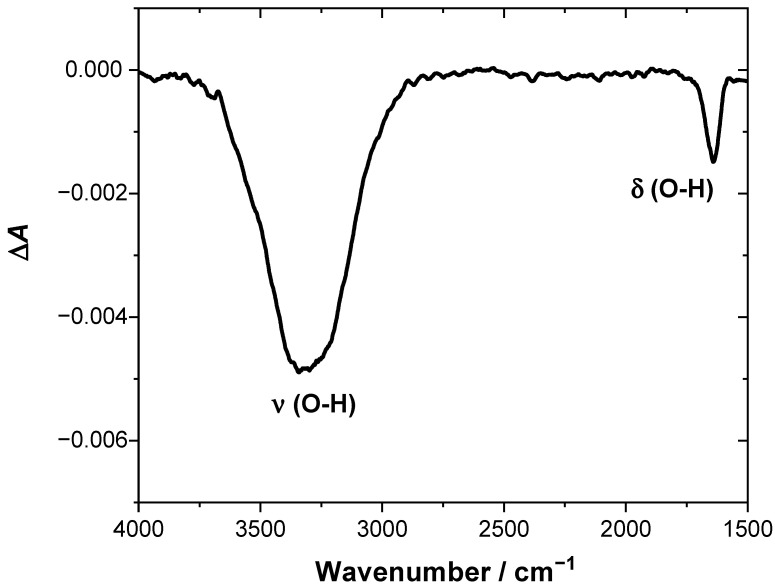
The SEIRA spectra representing the difference in the absorbance between the sample spectrum recorded after one freeze–thaw cycle and the reference spectrum collected before the freeze–thaw cycle. Negative *v*(O–H) and δ(O–H) bands are indicative of loss of water at the electrode–electrolyte interface.

The results obtained for the lipid film before freeze–thaw treatment demonstrate that membrane resistance varies only slightly with potential and its value is within the range of 170–200 kΩ cm^2^. Based on AFM data, we may assume that the permeability of this membrane will be strongly affected by the presence of numerous pinholes, which facilitate uncontrolled ion and water flux across the membrane. The latter contributes to the relatively high ionic conductivity of the membrane, which determines the membrane resistance. In contrast, the *R*_m_ for lipid film after freeze–thaw treatment varies noticeably with the potential, and its numerical values are significantly higher. Between the potentials of +0.2 V and −0.1 V where the lipid film is most stable, the membrane resistance is ~1.0 MΩ cm^2^, which is a manifestation of good insulating properties and low permeability for ions and water molecules. As demonstrated by AFM data, the lipid film undergoes reorganization upon freeze–thaw treatment. The initially formed defected double bilayer is transformed into the virtually defect-free single bilayer with some patches of the double bilayer. The substantially decreased density of defect sites hinders the ionic transport across the membrane, hence its conductivity is decreased. Consequently, the membrane resistance becomes high. More negative polarization of the electrode results in a gradual decrease of the resistance, which might be related to the onset of the electroporation process and/or accumulation of water and ions at the interfacial region typically observed for lipid membranes at the negative potentials [31,41]. Finally, at the potential of −0.4 V, the membrane resistance becomes comparable to that observed for lipid film before the freeze–thaw cycle, indicating that defect density is similar for both systems within that potential range. Comparison of the data indicates clearly that the insulating properties of the lipid film assembled from bicelles are improved upon freeze–thaw treatment. This is also confirmed by the SEIRAS results. Figure 7 shows the SEIRA spectra, which represents the difference in absorbance between the sample spectrum recorded after one freeze–thaw cycle and the reference spectrum recorded before thermal treatment. It demonstrates that freeze–thaw treatment leads to loss of water from the electrode–electrolyte interface, as can be concluded from the presence of the negative *v*(O–H) and δ(O–H) bands. This effect can be explained by the sealing of the pinholes and reorganization of the lipid film, which leads to the removal of the water molecules from defects. Additionally, the diffusion of water entrapped between the lamellae into the bulk phase may also contribute to the net loss of water, which will be discussed in more detail below. It should also be mentioned that the SEIRA spectrum in Figure 7 does not contain any bands related to *v*(C–H) and *v*(C=O) vibrations from lipids. This suggests that the amount of the lipidic material deposited on the electrode surface remains unchanged upon thermal treatment.

**Figure 8:**
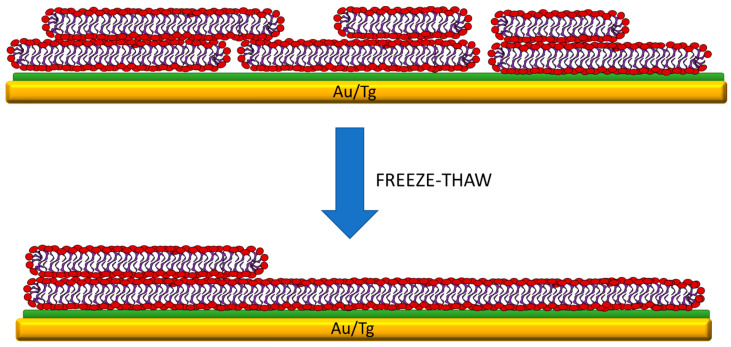
Model illustrating spontaneous deposition of the bicelles on the thioglucose-modified gold electrode and the effect of the freeze–thaw treatment on the structure of the lipid film. Spontaneous deposition of bicelles leads to the formation of a defected double bilayer, but the freeze–thaw treatment causes reorganization of the membrane structure into a defect-free single bilayer with the locally occurring double bilayer.

Based on our experimental observations, we propose the possible scenario of bicelles deposition on thioglucose-modified gold electrode and the transformation of the resulting film into a single bilayer, which is depicted in Figure 8.

Spontaneous deposition of bicellar mixture onto the thioglucose-modified gold electrode results in the formation of the double bilayer with a relatively large number of pinholes. The latter facilitates uncontrolled ion and water transport from the bulk of the solution to the surface of the electrode and contributes to the low resistance of the membrane. Since the double bilayer is a case of a multilamellar system, it means there are two pools of water, i.e., interlamellar water including molecules entrapped between two bilayers as well as water separating the bottom bilayer from the electrode, which can be considered as one pool, and bulk water which can be considered as a second pool. Importantly, these two pools of water have different freezing characteristics. As it was demonstrated for multilamellar lipid vesicles, the interlamellar water can be supercooled to approximately −40 °C before it freezes by homogeneous nucleation [44]. We believe this feature is crucial for the observed reorganization of the membrane. During the freezing cycle, when the temperature is lowered below 4 °C, the ordering of the hydration shell of the top bilayer is expected to decrease and the distance between polar heads is increased [38]. This produces certain asymmetry, which may favor the rearrangement of the molecules to minimize the energy of the system. Further decrease of the temperature causes freezing of the bulk water, while the interlamellar water is liquid, which means that the top membrane experiences the osmotic and mechanical stress, resulting in a diffusion of water molecules to the bulk water phase, and consequently the lamellarity of the film can be decreased similarly as it is observed for the transition from multilamellar to unilamellar vesicles [44,45]. This process is accompanied by the sealing of the pinholes since the lipid material from the top leaflet does not desorb from the electrode surface, but it is redistributed.

## 4. Conclusions

In this work, we have evaluated the properties of the lipid membranes obtained by adsorption of bicelles on thioglucose-modifed gold electrodes. We have demonstrated that adsorption of bicelles occurs by the replacement of the interfacial water. Their spontaneous deposition leads to the formation of a double bilayer structure on the electrode surface. However, the resulting lipid assembly contains numerous defects and pinholes which span either the entire thickness of the film or only the top bilayer. This in turn, affects the electrochemical properties of the membrane, which was found to be permeable for ions and water. Significant improvement in terms of the morphology of the lipid film and its electrochemical characteristics is achieved upon freeze–thaw treatment. The number of pinholes and defect sites is reduced, and the lipid assembly is rearranged to a single bilayer configuration with locally occurring patches of the second bilayer on top of it. Electrochemical characterization of the lipid membrane after freeze–thaw treatment demonstrated that its permeability for ions and water is significantly reduced. The latter is manifested by the relatively high value of the membrane resistance of ~1 MΩ cm^2^. We believe that the approach presented in this work offers an alternative way to obtain stable planar lipid membranes with good insulating properties. In perspective, the benefit of such an approach is that bicelles are known to be a suitable lipid environment for reconstitution and studies of the transmembrane proteins. Hence, more complex cell membrane mimics can be obtained by depositing bicellar mixtures on electrodes.

## Supplementary Materials

The following are available online at https://www.mdpi.com/2077-0375/11/1/11/s1, Figure S1: AFM image of a bare Au(111) electrode collected in an aqueous solution of 0.1 M NaF. The image was taken in PeakForce Tapping mode. Figure S2: AFM image and cross-sectional profile of the ripple phase. The image was collected in an aqueous solution of 0.1 M NaF using PeakForce Tapping mode.
