# Supplementary material for: Electrochemical Properties of Lipid Membranes Self-Assembled from Bicelles
Source: Membranes (Basel). 2020 Dec 23;11(1):11. doi: 10.3390/membranes11010011 (PMC7824464; doi:10.3390/membranes11010011)
Supplement: Supplementary file 1 [file membranes-11-00011-s001.pdf]

membranes

Supporting Material

Electrochemical Properties of Lipid Membranes Self-Assembled from Bicelles

Damian Dziubak [1,*], Kamil Strzelak [2] and Slawomir Sek [1]

[1] Faculty of Chemistry, Biological and Chemical Research Centre, University of Warsaw, Żwirki i Wigury 101, 02-089 Warsaw, Poland; slasek@chem.uw.edu.pl
[2] Faculty of Chemistry, University of Warsaw, Pasteura 1, 02-093 Warsaw, Poland; kamil.strzelak@chem.uw.edu.pl
* Correspondence: ddziubak@chem.uw.edu.pl

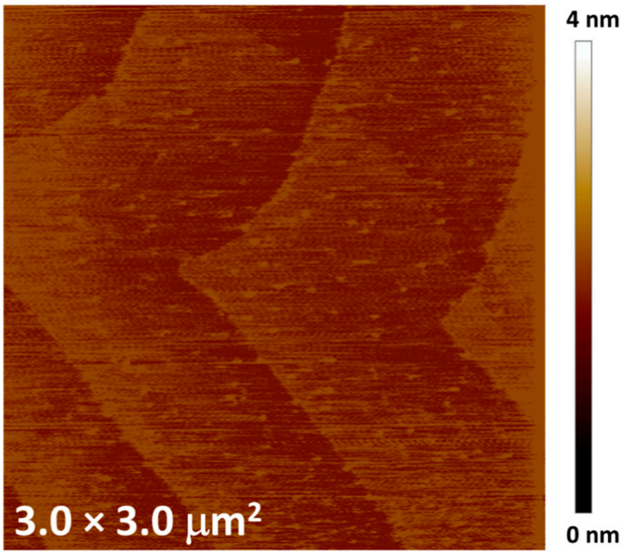

Figure S1. AFM image of bare Au(111) electrode collected in an aqueous solution of 0.1 M NaF. The image was taken in PeakForce Tapping mode.

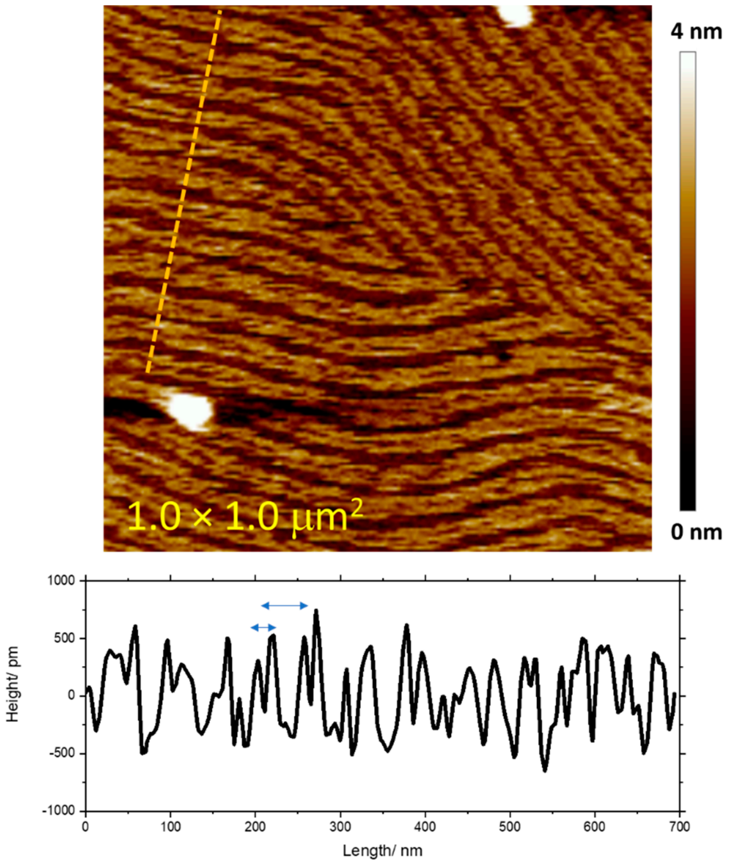

Figure S2: AFM image and cross-sectional profile of the ripple phase. The image was collected in an aqueous solution of 0.1 M NaF using PeakForce Tapping mode.
